# #Deathbedlive: the end-of-life trajectory, reflected in a cancer patient’s tweets

**DOI:** 10.1186/s12904-018-0273-9

**Published:** 2018-01-22

**Authors:** Joanna Taylor, Claudia Pagliari

**Affiliations:** 1 eHealth Research Group, Usher Institute of Population Health Sciences and Informatics, University of Edinburgh, Edinburgh, UK; 2 Ernst and Young AG, Basel, Switzerland

**Keywords:** Cancer, Palliative care, End-of-life care, Death and dying, Social media, Twitter, Patient experience, Illness trajectories, Digital ethnography, Netnography

## Abstract

**Background:**

Understanding physical and psycho-social illness trajectories towards the end of life can help in the planning of palliative and supportive care. With terminal patients increasingly seeking and sharing health information and support via social media, it is timely to examine whether these trajectories are reflected in their digital narratives. In this exploratory study, we analysed the Twitter feed of prominent cancer sufferer and physician, Kate Granger, over the final 6 months of her life.

**Methods:**

With the consent of Kate’s widower, Chris Pointon, 1628 Twitter posts from @GrangerKate were manually screened. The 550 tweets judged relevant to her disease were qualitatively content analysed with reference to the six modifiable dimensions of the patient experience in Emanuel and Emanuel’s ‘framework for a good death’. The frequency of each tweet category was charted over time and textual content was examined and cross-referenced with key events, to obtain a deeper understanding of its nature and significance.

**Results:**

Tweets were associated with physical symptoms (*N* = 270), psychological and cognitive symptoms (*N* = 213), social relationships and support (*N* = 85), economic demands and care giving needs (N = 85), hopes and expectations (*N* = 51) and spiritual beliefs (*N* = 7). While medical treatments and procedures were discussed in detail, medical information-seeking was largely absent, likely reflecting Kate clinical expertise. Spirituality was expressed more as hope in treatments or “*someone out there listening*”, than in religious terms. The high value of Kate’s palliative care team was a dominant theme in the support category, alongside the support she received from her online community of fellow sufferers, friends, family and colleagues. Significant events, such as medical procedures and hospital stays generated the densest Twitter engagement. Transitions between trajectory phases were marked by changes in the relative frequency of tweet-types.

**Conclusions:**

In Kate’s words, *“the power of patient narrative cannot be underestimated”*. While this analysis spanned only 6 months, it yielded rich insights. The results reflect theorised end-of-life dimensions and reveal the potential of social media data and digital bio-ethnography to shine a light on terminal patients’ lived experiences, coping strategies and support needs, suggesting new opportunities for enhancing personalised palliative care and avenues for further research.

## Background

In 1968 Glaser and Strauss described the advancement towards death as having the elements of time and shape [1] giving rise to the concept of the illness trajectory. Originally developed to describe how physical aspects of a patient’s disease unfold through the phases of pre-trajectory, trajectory onset, living with disease progression, downward phase and dying [2], the concept has since been expanded to include psychosocial aspects of the patient experience, including their response to their illness, the people around them and the interventions undergone [3]. Emanuel and Emanuel’s ‘framework for a good death’ [4] has been particularly influential in helping clinicians to better anticipate the needs of patients during the progression of their illness and to shape palliative care services. The Framework articulates six ‘modifiable dimensions’ of the patient experience related to 1) physical symptoms, 2) psychological and cognitive symptoms, 3) social relationships and support, 4) economic demands and care giving needs, 5) hopes and expectations, and 6) spiritual and existential beliefs.

**Fig. 1:**
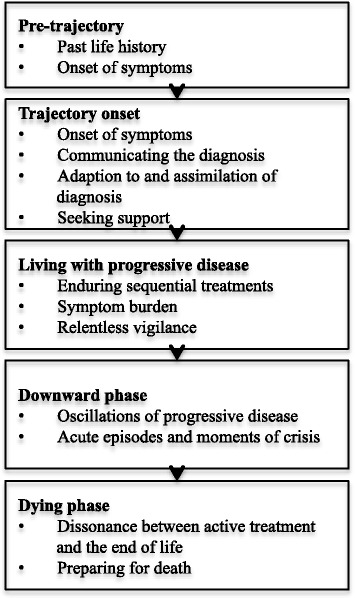
Cancer illness trajectory phases [34]

Most research into illness trajectories originates from the fields of public health and social sciences, drawing on studies using qualitative or mixed-methods, with data typically gathered from cohorts of patients through focus groups, surveys and interviews. This research has revealed different illness trajectories for different terminal conditions, with the cancer trajectory described as a steady progression over a period of weeks-and sometimes years, punctuated with the positive and negative effects of oncology treatment, weight loss, reduction in physical performance and the impaired ability to self-care during the last few months, as shown in Fig. 1 [3]. Cancers can also have unique trajectories, depending on issues such as prognosis, pain, disfigurement and response to treatment [5] while the same type of cancer progression may be experienced differently as a consequence of personal and social factors such as resilience and availability of emotional support [6].

### Social media in health

Technological advances over the past decade have enabled widespread access to the Internet in most countries, with the use of social media sites becoming increasingly common. Social media are a class of online, often mobile, platforms that support the creation and exchange of user-generated content [7], a phenomenon sometimes referred to by the terms Web 2.0 or Social Web. Social media include generic platforms for networking, information sharing and collaboration (e.g. Facebook, Twitter, YouTube, LinkedIn), as well as online forums aimed at specific communities (e.g. PatientsLikeMe, Mumsnet). As health and wellness are dominant societal concerns, they account for a considerable amount of social media activity, and some analysts have referred to this as Health 2.0 or Medicine 2.0 [8].

Several uses of social media in research have been described in the literature. These include the deployment of social media platforms for the *conduct* of research, such as for online surveys [9], recruitment to studies [10] and participative ‘citizen science’ [11], and as a *source of data* for research [12]. The latter ‘secondary uses’ include social, business and policy research examining user demographics, interactions and networks, ‘social listening’ to understand sentiments associated with particular topics or products [13, 14] and ‘big data’ analytics to uncover new associations or predict future behaviours or outcomes. The term “infoveillance” has also been used to refer to the automated and continuous analysis of unstructured, free text information available on the Internet for the purpose of public health monitoring [15].

### Social media use by cancer patients

A scoping review of studies involving social media use by patients and caregivers [16] reported that discussion forums, online support groups, social networking sites and micro-blogs, such as Twitter, dominated the published literature, with 11.3% of the 284 identified studies focusing on cancer. Examples included a study examining the use of Twitter and its role in the lives of cancer patients, which revealed that that the majority of tweets focused on psychological support [17], and a narrative synthesis of cancer patient blogs, which indicated that users share their experiences online in order to find information, maintain relationships with others, inform their health behaviour, and ‘tell their story’ about their treatment, diagnosis and symptoms and their experiences of health services [18].

Historically death and dying has been seen as a taboo topic for public discussion, however the willingness to talk openly about it online by terminally ill patients, their families and palliative health and social care professionals, has increased over recent years giving rise to an emerging field of research [19]. The first empirical study to have analysed data from Twitter for understanding disease was published in 2010 [20], yet among the many subsequent studies using Twitter in such research [21], we have been unable to find any empirical studies that have analysed how the online activity of cancer patients reflects the illness trajectories documented in previous research or explored the potential of this emerging data source to yield insights about cancer patients’ experiences at the end-of-life.

### Aims of this study

This study sought to systematically analyse the content of one prominent cancer patient’s Twitter feed in the final 6 months of her life, in order to determine its fit with documented end-of-life trajectories and the 6 dimensions of the ‘framework for a good death’, as well as to explore the value of social media data for understanding patients’ personal experiences, life quality and coping strategies. The study was intended as exploratory and hypotheses-generating, with a view to providing insights to inform future research and the design of innovative palliative care services.

### Kate granger

Kate Granger was an English geriatrician and campaigner for better patient care. In 2011 at the age of 29, she was diagnosed with a rare form of sarcoma, known as desmoplastic small-round-cell tumour, with a predicted to life expectancy of around 5 years. She was treated with P6 protocol chemotherapy and endured painful treatments, which she described in detail in her blogs “The Other Side and the Bright Side” [22]. Kate created her Twitter account (@GrangerKate) in March 2012 and, prior to her death on 23 July 2016, posted approximately 12,500 tweets and attracted approximately 48,000 followers [23].

Through her experiences as a patient, she and her husband founded the “#hellomynameis” campaign encouraging healthcare staff to introduce themselves to patients. They raised over £250,000 for local cancer charity the Yorkshire Cancer Centre Appeal and in 2015 she was awarded an MBE for her services to the British National Health Service (NHS). As a young woman familiar with social media, Kate’s story represents a valuable opportunity to examine the emergence and progression of a personal narrative, in the public domain, about coping with terminal illness.

## Methods

Although the cancer trajectory can last for years, and did so for Kate Granger, we chose to study the final 6 months of life, which represents the terminal phase and is commonly associated with preparation for death and the commencement of hospice services, for those fortunate enough to receive the latter [24]. Original tweets, re-tweets and responses posted between 1 January 2016 and 25 July 2016 using the account @GrangerKate, were manually extracted for categorization and analysis. The data extracted included the date and time of posting and the up to 140-character text contained within the tweet.

The tweets were then manually screened for their relevance to the disease, based on predefined inclusion and exclusion criteria, as described in Table 1.

**Table 1:** Tweet inclusion and exclusion criteria

Inclusion criteria: • Tweets posted by @GrangerKate’s Twitter account • Original tweets, annotated re-tweets and personal responses posted between 0:00:00 (UTC) 1 January 2016 and 23:59:59 (UTC) 25 July 2016 • Tweets that were considered directly relevant to the terminal condition
Exclusion criteria: • Tweets posted on other Twitter accounts • Simple re-tweets of other people’s postings (with no further annotation) • Tweets posted outside the timeframe indicated • Tweets that were considered not directly relevant to the condition, such as those associated with the “#hellomynameis” campaign, Kate Granger’s fundraising activities, her views on the NHS and politics as well as news media • Images or URLs embedded within tweets

Drawing on principles of digital ethnography [25] we used qualitative content analysis [26] to summarize, chart and interpret the eligible tweets. Tweet content was first categorised according to the six modifiable dimensions of the patient experience in the ‘framework for a good death’, shown in Table 2, with each post treated as a single unit of interaction and the categories as non-exclusive. The narrative content of tweets was also examined, to obtain further contextual information about significant events and personal responses. The frequency of each category, as well as the occurrence of key events (such as medical procedures or transfer to hospice) were plotted over time and converged with the qualitative data in order to “learn the meanings, norms, patterns of a way of life” ([27]: pg13) and to enable comparisons to be made with published end-of-life trajectories. Images and web links included in the tweets were not reviewed during the screening.

**Table 2:** Modifiable dimensions of the patient experience, from the ‘framework for a good death’ [4]

Modifiable dimension of the patient experience	Examples of specific concerns
Physical symptoms	Pain and fatigue
Psychological and cognitive symptoms	Depression, anxiety and confusion
Social relationships and support	Family, community, interests
Economic demands and care giving needs	Saving and income, personal care and nursing care
Hopes and expectations	Milestones and assessment of prognosis
Spiritual and existential beliefs	Religion, sense of purpose and meaning

### Ethical considerations

Although the data available on Twitter exist in the public domain and can therefore be mined for research purposes without the need to obtain explicit informed consent from the data subjects [28], ethical research conduct and digital etiquette are nevertheless required. Given the lack of consensus on ethical principles for the secondary use of social media data, we applied relevant sections from guidance developed by the UK Economic and Social Research Council (ESRC) [29], the British Psychological Society (BPS) [30] and the Association of Internet Researchers (AoIR) [31], as recommended by a recent review on the readiness of ethics guidelines to address this type of research [32]. While the ERSC “Framework for Research Ethics” cautions that studies involving online respondents may involve more than minimal risk [29], the BPS “Guidelines for ethical practice in psychological research online” go further, by differentiating between participants who are identifiable or anonymous and those who are actively recruited or studied without their knowledge [30]. The AoIR guidance on “Ethical Decision-Making and Internet Research” provides a series of questions for researchers to consider when conducting studies of this type, and reflect the context, risks and management of the data and how the findings will be presented [31].

In the case of this study, written agreement to extract, analyse and publish the tweets posted by @GrangerKate was sought and obtained from Kate Granger’s widower, Chris Pointon, via email.

## Results

Kate’s tweets were first screened for eligibility and those that were considered relevant to her condition were then classified and plotted on a visual timeline.

### Tweet eligibility

**Fig. 2:**
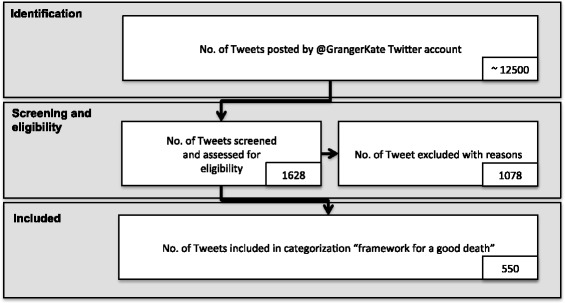
Steps in screening and assessing the tweets

Of the 1628 tweets posted by @GrangerKate during the 6-month period, 550 were considered relevant to her condition and therefore included in the classification, as described in Fig. 2. The remaining 1078 tweets were excluded for reasons such as their focus on her campaigning activities (388) as well as her views on the NHS (109) and politics (105), as summarised in Table 3.

**Table 3:** Number of tweets excluded and the reason for exclusion

Reason for exclusion	Number of tweets
Campaigns including “#hellomynameis” and fundraising activities	388
Professional work as a geriatrician	89
Hobbies including baking, flute and band practice	48
NHS in general	109
Politics including her support for the Junior Doctors’ strike	105
Dissemination of news and research	38
Others, including general “thank you” messages and topics that could not be determined from the tweet narrative	437

The 550 tweets considered relevant to the condition, were classified according to the modifiable dimensions of the patient experience in Emanuel and Emanuel’s ‘framework for a good death’. The total number of tweets classified for each dimension is summarized in Table 4, including an example of the tweet narrative in each category.

**Table 4:** Number of tweets per modifiable dimension

Modifiable dimension of patient experience	Number of tweets	Examples of tweets
Physical symptoms	270	“I am having a temporary nephrostomy this morning... With a view to then getting the radiotherapy done then performing a stent exchange” – 15 June 2016
Psychological and cognitive symptoms	213	“I’m not sure I can do this.” – 3 April 2016
Social relationships and support	85	“Everybody being so lovely both in public & behind the scenes on Twitter. Thank you so much. Love our virtual family very much” – 8 May 2016
Economic demands and care giving needs	85	“After seeing my lovely palliative care nurse this a.m. we’ve decided hospice admission for symptom control best course of action.” – 8 July 2016
Hopes and expectations	51	“Perhaps I should just accept #deathbedlive is closer than I hoped it was & get my final preparations finished.” - 29 March 2016
Spiritual and existential beliefs	7	“A comfortable night, just one would be so appreciated if anyone is listening. Running on empty and a nonstop few days coming up.” – 3 March 2016

### Nature of tweets over time

**Fig. 3:**
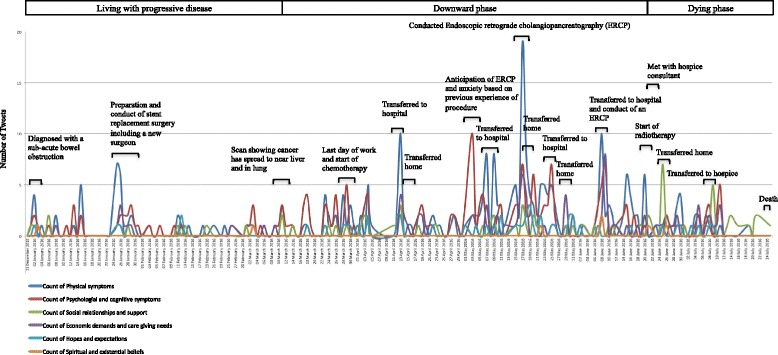
Kate Granger’s illness trajectory

Figure 3 visualises Kate Granger’s digital end-of-life trajectory. The coloured lines represent the frequency of daily tweets, according to each of the six dimensions of the ‘framework for a good death’, plotted over the 6-month observation period. Similar to Barclay et al.’s study into the trajectories to death in residential care homes [33], the annotations in Fig. 3 describe key contextual events and the superscript shows the broad phases of the illness trajectory, which were evident in Kate’s tweets.

Given that Kate had been diagnosed with cancer for 5 years, Corbin and Strauss’ pre-trajectory and trajectory onset phases were not represented in the sample of tweets. However, Tweet characteristics indicative of the living with progressive disease, downward trajectory and dying phases were evident and these form the basis of our findings.

### Living with progressive disease phase

The ‘living with progressive disease’ phase can in some cases last several years, where patients go through cycles of feeling and looking well and living life to the full, followed by periods of disease progression and illness. Although the full extent and duration of this phase was not analysed as part of this study, which focused on the final 6-months of life, initial findings indicate that Kate posted 600 tweets, from the beginning of the observation period on 1 January 2016 until 20 March 2016, of which only 16% were associated with her condition. She endured a series of sequential treatments and procedures including her seventh stent replacement in late January 2016, with the symptom burden being indicated by tweets such as “*started 2015 worrying about cancer. Start 2016 worrying about cancer*” and “*we've been living with the reality of cancer in our lives for nearly 5 years…it becomes the new normal*” – 1 January 2016.

Uncertainty seemed to pervade Kate’s life during this phase as indicated by her tweet “*In pain. Horrible achy pelvic pain….Are you waking up cancer or is it in my head?*” – 21 February 2016. She was at home and still able to work, requiring minimal caregiver support and continued with her hobbies and campaigning activities. She oscillated between disease progression and treatment, with psychological and cognitive symptoms appearing to closely follow the physical symptoms. She had received support from a palliative care team from an early stage (“*Not been to hospice yet but had palliative care team support almost from day one…” –* 4 May 2016). Although treatment allowed some respite from symptoms of progressive disease (“*It kept me well for nearly 6 months. But was never going to cure me & was always a palliative measure…*” – 13 March 2016), living with the condition appeared exhausting in its relentlessness (“*I'm just so tired of being in pain. Nagging pain that I can't escape night after night. Had enough.”* – 21 April 2016).

### Downward phase

Kate’s downward phase was characterized by increasingly frequent episodes of illness and crisis over a 4-month period between 20 March 2016 and 21 June 2016, which is reflected in a marked increase in the frequency of her tweets. Peaks in this digital trail marked acute episodes, associated with treatments and procedures such as chemotherapy, radiotherapy and endoscopic retrograde cholangiopancreatographies (ERCP), and four stays in hospital over a 10-week period. By this stage of her illness she was no longer able to work and many of her tweets detailed the physical and psychological anxiety she experienced as well as the support provided by healthcare professionals and by her physical and virtual community of family, friends and followers. During this phase she posted 898 tweets of which 42% were associated with her condition.

During these acute episodes, tweets conveyed a lack of confidence in being able to cope (“*I'm exhausted of being 'brave', the expectation that I'm 'Dr Kate Granger' and therefore can cope with anything. I can't...*” – 3 May 2016) and acknowledging that the end was near (“*Perhaps I should just accept #deathbedlive is closer than I hoped it was & get my final preparations finished.” –* 19 July 2016)*.* Respite came from being at home (“*Very happy to be on the way home. Thank you so much to #nhs for scraping me up & putting me back together…”* – 18 May 2016) and the positive messages of thanks and support given by those around her (“*Aww having a little smiley weep at all the wonderful messages. Everyone is just so kind. Thank you so much xx #kateshomecoming*” – 25 June 2016).

### Dying phase

In the 4-weeks, prior to her death on 23 July 2016, Kate experienced a rapid decline in health, which was mirrored by a decline in the number of tweets posted, down to 130 of which 57% were associated with her condition*.* Kate’s acknowledgement of the terminal phase is indicated in her first reference to the transition to hospice care, with the tweet “*Hospice waiting list was going to be well into next week so decided to bite the bullet and come home…”* – 23 June 2016. She appeared to be preparing for death during the 4-week period after meeting with the hospice consultant on 29 June 2016, before being transferred to St Gemma’s Hospice on 8th July. In contrast to the previous phases, only 30% of Kate’s Tweets in the dying phase referred to her physical and psychological symptoms, and the remainder instead focused on the social and caregiving support she received from her palliative care and hospice team, and her family, friends and online followers.

## Discussion

We have described the pattern of physical, psychological, social and care needs of high profile sarcoma patient Kate Granger, as represented in the posts from her Twitter account over the 6-month period prior to her death. Our analysis indicates that the digital manifestation of cancer progression maps to the illness trajectories described in existing palliative care research and to the key dimensions of the patient experience articulated in Emanuel and Emanuel’s ‘framework for a good death’. Our analysis has also tested the use of social media data and digital ethnographic approaches for exploring the lived experiences of patients nearing the end of life.

### Parallels between the digital end-of-life trajectory and existing models

In interpreting the digital trajectory seen in Kate Granger’s Twitter postings, we drew on the general cancer trajectory described in the literature, in which patients experience an onset of incurable disease and a short and rapid decline over a few months. This type of trajectory involves a reasonably predictable decline in physical health, with progression punctuated by the positive and negative effects of palliative oncology treatments [3]. While our analysis focuses on the later stages of the cancer illness trajectory it is important to consider the similarities and differences between the patterns seen in Kate Granger’s tweets and these general trajectory descriptions. In Kate’s case, similarities can be drawn in relation to her endurance of sequential treatments, the acute episodes and moments of crisis as well as her preparations for death. As noted in the introduction, the trajectory of different cancers varies, to some extent, and it is important to bear this in mind when seeking to interpret the patterns observed in individual cases. For example, Reed and Corner’s research into the illness trajectory of metastatic breast cancer identified a “rollercoaster” trajectory, where the typical duration was 2-5 years and patients, similarly to Kate Granger, experience oscillations of disease progression, treatment and restoration of well-being, as well as acute crisis episodes [34].

Emanuel and Emanuel’s ‘framework for a good death’ proved a useful tool for classifying the data, which showed a good fit with their six ‘modifiable dimensions of the patient experience’, and helped in the plotting of physical symptoms and psychosocial responses. For example, the differentiation of tweet types aided the interpretation of changes in Twitter activity between the different phases of the illness trajectory, such as the priority accorded to physical and medical issues in the earlier stages, compared to the focus on people and comfort-giving at the end.

It is interesting to note that, based on her tweets over the 6-month period, Kate made no explicit reference to spirituality, which contrasts with previous observations in palliative care research [35]. Despite this, there were frequent references to metaphysical concepts, such as the call for ‘someone out there’, suggesting that existential concerns may simply be taking new forms with the decline of traditional religious practices in UK society.

Also absent in Kate’s Twitter narrative are ‘battle metaphors’, such as talk of fighting or being at war with cancer. While these are common in some cultural and clinical settings and have been the subject of research [36], experts have advised healthcare professionals against using them with patients, to avoid inducing feelings of failure for what is a biologically-determined outcome [37]. As a health professional, Kate Granger would have been acutely aware of her prognosis and may thus have chosen to focus on coping and preserving her quality of life. This also illustrates the need to recognise that individual patient characteristics can influence how a ‘good death’ is experienced [4].

### Benefits of analysing social media data

This modest study takes a first step in demonstrating how these emerging data sources may elucidate terminal patients’ physical and psychosocial responses during the illness trajectory and thus help to inform the provision of supportive and palliative care services at different stages. Analysing the social media postings of individuals like Kate Granger can provide a unique window into their ‘lived experiences’, including at highly emotional and sensitive stages, which can be difficult to access using conventional direct research methods. Social media are becoming the norm for communication amongst younger people and as these ‘digital natives’ progress to later stages of life, where death becomes more imminent, there are likely to be greater opportunities for such research.

Social media data are not only useful for focused studies, as we have undertaken here, but also present opportunities for research at scale. Automated tools for social media mining, natural language processing and sentiment analysis – such as those from Crimson Hexagon, Hootsuite, Symplur, Keyhole and Sproutsocial - are now widely used in the marketing sector and are transferable to academic research [38]. Other forms of digital data are already being used in this way. For example, in the only previous study we found to have charted the cancer trajectory for sarcoma, Tang and colleagues converged electronic records with individually-administered questionnaires, in order to profile levels of distress in as many as 74 patients before, during and after surgery, with the aim of understanding the psychological and socioeconomic factors influencing resilience, coping and outcomes [5]. A study currently underway at Stanford University [39] is using historical patient data to train Deep Learning algorithms to identify dying patients from their electronic health records and proactively bring them to the attention of palliative care staff in a hospital setting. Given such developments, it is not unreasonable to envisage a future in which data from patients’ social media feeds, healthcare records and wearable monitoring devices are linked and processed using artificial intelligence, to generate real-time adaptive decision support for ‘precision’ palliative care.

### Implications for supportive and palliative care

Illness trajectories have proven valuable as a means of describing the physical and psychosocial progression of cancer and other conditions [3]. While mapping the objective physical and medical aspects of these journeys is relatively straightforward, mapping their psychological and socio-emotional aspects typically requires in-depth qualitative studies with patients and their loved ones, which limits the usefulness of the findings for practitioners. Being able to study the journey towards death in the digital world opens a new window into the concerns, needs and vulnerabilities dying patients experience at different points in time, which may help to target the provision of supportive and palliative care, as well as enabling health professionals to understand patients’ perspectives on the care they deliver.

The longitudinal data posted by Kate Granger provide evidence of the involvement of palliative care services during the patient journey. In Kate’s case, she received palliative care relatively early, with her first tweet about this being posted on 25 October 2012, 14 months after her initial diagnosis. This is later supported by her tweet *“…had palliative care team support almost from day one. They've been amazing... See the whole of me*” – 4 May 2016. While palliative care services still tend to focus on the shorter dying phase [34] research has demonstrated that engagement over longer trajectories can enable better advanced planning for a good death, empower patients attempting to gain control over their illness and help to alleviate concerns about the possible nature of death [3]. All of these were evident in Kate’s tweet “*After seeing my lovely palliative care nurse this a.m we've decided hospice admission for symptom control best course of action..*.” - 8 July 2016.

End-of-life care planning must be multi-dimensional, with palliative care services playing the role of a mediator in helping patients to cope with their illness, optimising quality of life and achieving a dignified and peaceful death. Previous studies, including those focused on sarcoma patients, have revealed a range of problem-focused coping strategies, such as information seeking, choosing one’s treatment team, and advocacy for oneself, as well as emotion-focused strategies such as support seeking, present-moment focus, distraction, denial and oversleeping [6]. Future analyses of patients’ social media activity may help to verify or shed further light on these strategies, in addition to profiling illness trajectories and dimensions of the ‘good death’ framework. It may also help practitioners to better understand differences in patients’ responses to their illness. For example, based on our analysis of Kate Granger’s data, it appears that she did not use Twitter as a problem-oriented coping strategy, which might be explained by the focus on the last 6-months of her life rather than the period of initial diagnosis, or by her professional role as a geriatrician, which put her in a more informed role than most other patients. Understanding patients’ trajectories of need can also help palliative care professionals to better anticipate and proactively mitigate distress [35]. In Kate’s case this need for responsive approaches was evident in tweets such as “*Amazing care from #NHS today with my port flush, blood tests & psychology appointment…*” - 26 February 2016 and “*Psychology appt could not have come at a better time*” – 18 March 2016.

Another way in which digital ethnographic research methods may complement existing illness trajectory research, is by revealing the additional support provided to patients by their online social networks and communities. Kate Granger had many online followers, who helped to lift her spirits during periods of difficult treatment and distress, and studying the patterns of support and reciprocity in these digital spaces may suggest new ways in which to help patients nearing death. Despite Kate’s predicament, she did not appear to be lonely, as can often be the case, and regularly thanked her followers for their support during acute episodes of crisis. As such, palliative care teams may consider recommending that terminal patients establish an online presence on social media and share their experiences with others, both as a therapeutic coping strategy and as a means of obtaining additional social support beyond their immediate family, friends and care services. Of course, it should be recognised that not all patients are able or willing to share their experiences online and many prefer to remain anonymous when doing so.

Social media also offer opportunities to study patients’ *after* death. Services are emerging which enable users to preserve their digital legacy [40] through, for example, a social media auto-biography (e.g. deadsocial.org) or an avatar that draws on their social media history to engage in realistic interactions with friends and relatives (lifenaught.com) [41]. Although such tools may offer therapeutic value for patients preparing for a good death, or their bereaved loved ones, evidence of them doing so remains absent. This also raises new ethical and sociological questions about the responsible management of personas after death and the donation of social media archives for future research, in a similar way to the donation of medical records, tissue samples or body parts [42].

### Limitations

The cohort size of one limits the extent to which the findings of this study can be generalised to other patient groups. We recommend further comparable research to extend the evidence-base, including studies exploring whether the end-of-life trajectories reported for different conditions in previous research are also evident on Twitter or other social media platforms [35].

During our study, we did not extract and analyse the entire sample of 12,500 tweets from the 4 years that the @GrangerKate Twitter account was active; limiting the conclusions drawn with regards to the complete illness trajectory from diagnosis to death. Aside from further manual analysis, this could be addressed using automated social media mining techniques and natural language processing tools. However, these approaches also have challenges, including variability in the types of social media to which they are suited, the scope and quality of the data available for analysis, and the cost of software and data access licences.

Despite numerous studies into what constitutes a good death, there is little agreement about its definition. Key features have been identified as: preferences for a specific dying process, pain-free status, religiosity/spiritualty, emotional well-being, life completion, treatment preferences, dignity, family, quality of life and relationships with healthcare providers [43]. Although valuable and well respected, the ‘framework for a good death’ goes only some way towards accounting for the complexity of the end-of-life experience. In this study, the different dimensions were linked and therefore in some cases were difficult to distinguish as part of the analysis.

As already noted, the ethics of using potentially sensitive personal data placed on social media by individuals experiencing illness also represents a grey area, given the public nature of platforms like Twitter. Nevertheless, researchers should adhere with appropriate ethics guidelines, seek consent where appropriate and possible, and manage extracted data in a responsible and respectful way [44].

## Conclusion

We believe this is the first study to have systematically analysed the end-of-life illness trajectory expressed in a patient’s social media activity. Our results indicate that the data posted by terminal patients on Twitter can provide insights that may be comparable to, or compliment, those garnered using more traditional qualitative research techniques. To quote one of Kate’s tweets, *“the power of patient narrative cannot be underestimated”* – 13 April 2016. While our analysis was at the structured end of the digital ethnographic spectrum, it nevertheless shows the value of such methods for understanding how terminal disease is experienced by and affects individuals, how they cope, how support is sought and obtained and how patients feel about the ability of palliative care services to meet their needs at different stages. Further research is warranted to extend this analysis across the wider trajectory of life-limiting illness and to a variety of disease types, as well as to explore the use of data mining and pattern recognition techniques to study larger cohorts and different social media platforms. As part of a wider agenda for ‘palliative social media’ [19] we recommend efforts to engage health professionals in exploring how digital end-of-life trajectories may inform the provision of supportive and palliative care, to improve the quality of life and death for patients like Kate.

## Abbreviations

AoIR: Association of Internet Researchers
BPS: British Psychological Society
ERCP: Endoscopic Retrograde Cholangiopancreatographies
ESRC: Economic and Social Research Council
NHS: National Health Service

## References

[1] Glaser BG and Strauss AL. Time for dying. Chicago: Aldine Pub. Co.; 1968.

[2] Strauss LA, Glaser BC. Chronic illness and the quality of life. St. Louis: C.V. Mosby; 1975.

[3] Murray SA, Kendall M, Boyd K, Sheikh A (2005). Illness trajectories and palliative care. BMJ.

[4] Emanuel EJ, Emanuel LL (1998). The promise of a good death. Lancet (London, England).

[5] Tang MH, Castle DJ, Choong PFM. Identifying the prevalence, trajectory, and determinants of psychological distress in extremity sarcoma. Sarcoma. 2015;2015:745163. Epub 2015 Feb 12. https://www.ncbi.nlm.nih.gov/pmc/articles/PMC4342175/. 10.1155/2015/745163 PMC4342175 25767410

[6] Segal K, DuHamel KN, Posh L. Psychological Adaptation, Coping, and Distress in Adult-Onset Soft Tissue Sarcomas. ESUN A Periodical for the Sarcoma Community. 2010. http://sarcomahelp.org/coping.html.

[7] Kaplan A, Haenlein M (2010). Users of the world, unite! The challenges and opportunities of social media. Business Horizons.

[8] Van De Belt TH, Engelen LJ, Berben SA, Schoonhoven L (2010). Definition of health 2.0 and medicine 2.0: a systematic review. J Med Internet Res.

[9] Hilyard K, Broniatowski D, Dredzde M. How Far Can Twitter Reach in Good Survey Research? Social Science Space. 2015. https://www.socialsciencespace.com/2015/04/how-far-can-twitter-reach-in-good-survey-research/. Accessed 19 Jan 2018.

[10] Pedersen ER, Kurz J. Using Facebook for health-related research study recruitment and program delivery. Current Opinion in Psychology. 2016;9:38. 10.1016/j.copsyc.2015.09.011 PMC4697271 26726313

[11] Zooniverse. https://www.zooniverse.org.

[12] Wilson RE, Gosling SD, Graham LT (2012). A review of Facebook research in the social sciences. Perspect Psychol Sci.

[13] Anstead N, O'Loughlin B (2015). Social media analysis and public opinion: the 2010 UK general election. J Comput-Mediat Commun.

[14] Murphy J, et al. Social Media in Public Opinion Research: Report of the AAPOR Task Force on Emerging Technologies in Public Opinion Research. American Association for Public Opinion Research. 2014. https://www.aapor.org/AAPOR_Main/media/MainSiteFiles/AAPOR_Social_Media_Report_FNL.pdf. Accessed 19 Jan 2018.

[15] Eysenbach G (2009). Infodemiology and Infoveillance: framework for an emerging set of public health informatics methods to analyze search, communication and publication behavior on the internet. J Med Internet Res.

[16] Hamm MP, Chisholm A, Shulhan J, Milne A, Scott SD, Given LM, Hartling L. Social media use among patients and caregivers: a scoping review. BMJ Open. 2013;3(5). 10.1136/bmjopen-2013-002819 PMC3651969 23667163

[17] Gualtieri L, Akhtar FY (2013). Cancer patient blogs: how patients, clinicians, and researchers learn from rich narratives of illness. Information technology interfaces (ITI), proceedings of the ITI 2013 35th international conference on: 24-27 June 2013.

[18] Tsuya A, Sugawara Y, Tanaka A, Narimatsu H (2014). Do cancer patients tweet? Examining the twitter use of cancer patients in Japan. J Med Internet Res.

[19] Taubert M, Watts G, Boland J, Radbruch L (2014). Palliative social media. BMJ Support Palliat Care.

[20] Bernardo TM, Rajic A, Young I, Robiadek K, Pham MT, Funk JA (2013). Scoping review on search queries and social media for disease surveillance: a chronology of innovation. J Med Internet Res.

[21] Charles-Smith LE, Reynolds TL, Cameron MA, Conway M, Lau EHY, Olsen JM, et al. et al. Using social Media for Actionable Disease Surveillance and Outbreak Management: a systematic literature review. PLoS ONE 10(10):e0139701. doi:10.1371/journal.pone.0139701/. 10.1371/journal.pone.0139701 PMC4593536 26437454

[22] Kate Granger [https://en.wikipedia.org/wiki/Kate_Granger]. Accessed 19 Jan 2018.

[23] @GrangerKate [https://twitter.com/grangerkate?lang=en]. Accessed 19 Jan 2018.

[24] UK Department of Work and Pensions IB204. 2004. https://www.gpnotebook.co.uk/simplepage.cfm?ID=x20050803153747160230.

[25] Varis PK. Digital Ethnography. Tilburg Papers in Culture Studies Paper 104 (online) Tilburg University. 2014. https://www.tilburguniversity.edu/upload/c428e18c-935f-4d12-8afb-652e19899a30_TPCS_104_Varis.pdf.

[26] Hsieh H-F, Shannon SE (2005). Three approaches to qualitative content analysis. Qual Health Res.

[27] Hymes DH (1996). Ethnography, linguistics, narrative inequality : toward an understanding of voice.

[28] Hookway N (2008). Entering the blogosphere': some strategies for using blogs in social research. Qual Res.

[29] Economic and Social Research Council (UK) Framework for Research Ethics. 2015. http://www.esrc.ac.uk/files/funding/guidance-for-applicants/esrc-framework-for-research-ethics-2015/.

[30] British Psychological Society. Report from the Working Party on Conducting Research on the Internet. Guidelines for ethical practice in conducting psychological research online. http://www.bps.org.uk/sites/default/files/documents/conducting_research_on_the_internet-guidelines_for_ethical_practice_in_psychological_research_online.pdf.

[31] Markham A, Buchanan E. Ethical Decision-Making and Internet Research: Recommendations from the AoIR Ethics Working Committee. Association of Internet Researchers. 2012. http://www.aoir.org/reports/ethics2.pdf.

[32] Taylor J, Pagliari C. Mining social media data: how are research sponsors and researchers addressing the ethical challenges? Research Ethics 2017:1747016117738559. https://doi.org/10.1177/1747016117738559.

[33] Barclay S, Froggatt K, Crang C, Mathie E, Handley M, Iliffe S, Manthorpe J, Gage H, Goodman C (2014). Living in uncertain times: trajectories to death in residential care homes. Br J Gen Pract.

[34] Reed E, Corner J (2015). Defining the illness trajectory of metastatic breast cancer. BMJ Support Palliat Care.

[35] Murray SA, Kendall M, Grant E, Boyd K, Barclay S, Sheikh A (2007). Patterns of social, psychological, and spiritual decline toward the end of life in lung cancer and heart failure. J Pain Symptom Manag.

[36] Southall D (2012). The patient’s use of metaphor within a palliative care setting: theory, function and efficacy. A narrative literature review. Palliat Med.

[37] Gajewski M. May I take your metaphor? - how we talk about cancer. Cancer Research UK Science Blogs. 2015. http://scienceblog.cancerresearchuk.org/2015/09/28/may-i-take-your-metaphor-how-we-talk-about-cancer/.

[38] Mindruta R. Top 15 Free Social Media Monitoring Tools. Brandwatch, Blog. 2017. https://www.brandwatch.com/blog/top-10-free-social-media-monitoring-tools/.

[39] Avati A, Jung K, Harman S, Downing L, Ng A, Shah NH, Improving Palliative (2017). Care with deep learning.

[40] Cronin G. Living and dying on social media. CILIP Update. 2015. p. 35–37. http://www.cilip.org.uk/blog/living-dying-social-media.

[41] Calvard J. Death and Grief Online - The Opportunities and Challenges of Incorporating Digital Legacies into Palliative Care in Hospice Settings. St. Columba's Hospice. 2016. http://www.stcolumbashospice.org.uk/wp-content/uploads/2016/11/Digital-legacy-presentation.pdf.

[42] Shaw DM, Gross JV, Erren TC. Why You Should Donate Your Medical Data When You Die - Organs are not the only item of value from the deceased. Scientific American, The Conversation. 2017. https://www.scientificamerican.com/article/why-you-should-donate-your-medical-data-when-you-die/.

[43] Meier EA, Gallegos JV, Thomas LP, Depp CA, Irwin SA, Jeste DV (2016). Defining a good death (successful dying): literature review and a call for research and public dialogue. Am J Geriatr Psychiatry.

[44] Moorhead SA, Hazlett DE, Harrison L, Carroll JK, Irwin A, Hoving C. A new dimension of health care: systematic review of the uses, benefits, and limitations of social media for health communication. J Med Internet Res. 2013;15(4). 10.2196/jmir.1933 PMC3636326 23615206

